# Supplementary material for: Shotgun Metagenomic Sequencing Analysis as a Diagnostic Strategy for Patients with Lower Respiratory Tract Infections
Source: Microorganisms. 2025 Jun 9;13(6):1338. doi: 10.3390/microorganisms13061338 (PMC12196101; doi:10.3390/microorganisms13061338)
Supplement: Supplementary file 1 [file microorganisms-13-01338-s001.zip › microorganisms-3666346-supplementary.pdf]

Table S1. Performance of CDMs for BAL fluids

		Sensitivity	Specificity	Reference
Bacteria	MALDI-TOF MS	100% for ≥1×10^5 CFU/mL<br>79.2% for pure and dominant cultures	100%	[87]
	FA-PP	89.5-100%	95.3-99.8%	[88,89]
	TB/NTM PCR	82-100% for MTB<br>65% for NTM	98% for MTB<br>90-96% for NTM	[90]
	MTB/RIF assay	61.1-83.1% for MTB	84.1-96.6% for MTB	[91]
Fungi	MALDI-TOF MS	98.10%	100%	[92]
	P. jirovecii PCR	98.3%	91%	[93]
	Aspergillus antigen	88.6%	95.5%	[94]
	Cryptococcus antigen	93.1%	100%	[95]
Viruses	FA-PP	75-100%	97.1-100%	[88,89]
	CMV PCR	95%	98%	[96]

Abbreviations: CDM, conventional diagnostic method; BAL, bronchoalveolar lavage; MALDI-TOF MS, matrix-assisted laser desorption/ionization time-of-flight mass spectrometry; FA-PP, FilmArray® Pneumonia Panel; MTB, *Mycobacterium tuberculosis*; NTM, non-tuberculous mycobacteria; MTB, *Mycobacterium tuberculosis*; CMV, cytomegalovirus.
